# Supplementary material for: Reconciling Mining with the Conservation of Cave Biodiversity: A Quantitative Baseline to Help Establish Conservation Priorities
Source: PLoS One. 2016 Dec 20;11(12):e0168348. doi: 10.1371/journal.pone.0168348 (PMC5173368; doi:10.1371/journal.pone.0168348)
Supplement: S1 Dataset — (ZIP) [file pone.0168348.s002.zip › Taxa/Serra Norte/SN_2013/N4WS/Lista N4WS-17.pdf]

CAVIDADE N4WS-0017

Classe	Ordem	Fam/Outros	Gên/Outros	Espécie	S-U	U-S	Zon.
Annelida	Oligochaeta			jovem	1	1	E
Arachnida	Acari	(Oribatida)		jovem	X		
Arachnida	Acari	(Parasitengonina)		sp.1		X	P
Arachnida	Acari	(Parasitengonina)		sp.2		X	P
Arachnida	Acari	Ixodidae	*Amblyomma*	*cajennense*	X		
Arachnida	Acari	Rhagidiidae		sp.4		X	E
Arachnida	Acari	Trombidioidea		sp.	X		
Arachnida	Amblypygi	Phrynidae	*Heterophrynus*	*longicornis*	1	1	E
Arachnida	Araneae	Araneidae	*Alpaida*	sp.1	X	X	
Arachnida	Araneae	Barychelidae		jovem		1	E
Arachnida	Araneae	Ctenidae		jovem		1	E
Arachnida	Araneae	Ochyroceratidae	*Speocera*	sp.1		X	E
Arachnida	Araneae	Pholcidae	*Mesabolivar*	sp.	X	X	
Arachnida	Araneae	Salticidae		jovem		X	E
Arachnida	Araneae	Scytodidae	*Scytodes*	*eleonorae*	1		
Arachnida	Araneae	Scytodidae	*Scytodes*	*globula*		7	E/P
Arachnida	Araneae	Theraphosidae		sp.	X	4	E/P
Arachnida	Araneae	Theridiidae	*Dipoena*	sp.1		X	E
Arachnida	Araneae	Theridiidae	*Theridion*	sp.1	X	X	E
Arachnida	Araneae	Theridiosomatidae	*Plato*	sp.1	X	X	E/P
Arachnida	Araneae	Uloboridae		jovem		X	E
Arachnida	Opiliones	Escadabiidae		sp.2	X	X	P
Arachnida	Opiliones	Sclerosomatidae	Gagrellinae	sp.	X		
Arachnida	Palpigradi			jovem		X	P
Arachnida	Pseudoscorpiones	Chernetidae	*Spelaeochernes*	sp.1	X	X	E/P
Arachnida	Pseudoscorpiones	Chthoniidae	*Pseudochthonius*	sp.1		X	E
Arachnida	Pseudoscorpiones	Olpiidae		jovem		X	E
Chilopoda	Scolopendromorpha	Cryptopidae	*Cryptops*	sp.	X		
Chilopoda	Scolopendromorpha	Scolopocryptopidae	*Tidops*	sp.1		1	E
Diplopoda	Polydesmida	Fuhrmannodesmidae		jovem		X	P
Diplopoda	Polydesmida	Pyrgodesmidae		sp.1	X	X	
Entognatha	Collembola	Paronellidae		sp.1	X	X	E/P
Entognatha	Diplura	Campodeidae		sp.1		X	E
Insecta	Blattodea	Blaberidae		jovem		4	E
Insecta	Coleoptera	Carabidae		sp.20	X	X	E
Insecta	Coleoptera	Elateridae		sp.	X		
Insecta	Coleoptera	Staphylinidae		sp.62		X	E
Insecta	Diptera	Cecidomyiidae	Cecidomyiinae	sp.		X	E
Insecta	Diptera	Psychodidae	Phlebotominae	sp.		X	E/P
Insecta	Diptera	Psychodidae	*Pintomyia*	*gruta*	X		
Insecta	Diptera	Psychodidae	*Sciopemyia*	*sordellii*	X		
Insecta	Diptera	Sciaridae		jovem		X	E
Insecta	Diptera	Simuliidae		sp.		X	P
Insecta	Diptera	Tipulidae		sp.	X		
Insecta	Heteroptera	Cydnidae		jovem		X	P
Insecta	Heteroptera	Dipsocoroidea		sp.		X	E/P
Insecta	Heteroptera	Reduviidae	Reduviinae	sp.		6	E
Insecta	Heteroptera	Reduviidae	*Zelurus*	*festivus*	X		
Insecta	Heteroptera	Reduviidae	*Zelurus*	sp.	X		

Insecta	Hymenoptera	Bethylidae		sp.2		X	E
Insecta	Hymenoptera	Braconidae		sp.1		X	E
Insecta	Hymenoptera	Formicidae	*Gnamptogenys*	sp.1		X	E
Insecta	Hymenoptera	Formicidae	*Hypoponera*	sp.1		X	E
Insecta	Hymenoptera	Formicidae	*Hypoponera*	sp.2		X	E
Insecta	Hymenoptera	Formicidae	Myrmicinae	sp.1		X	E
Insecta	Hymenoptera	Formicidae	*Pachycondyla*	sp.2		X	E
Insecta	Hymenoptera	Platygastridae		sp.1		X	E
Insecta	Isoptera	Termitidae	*Nasutitermes*	sp.		X	E
Insecta	Isoptera	Termitidae	*Termes*	sp.		X	E
Insecta	Isoptera	Termitidae		sp.	X		
Insecta	Lepidoptera	Tineoidea		sp.	X	X	E
Insecta	Neuroptera	Myrmeleontidae		jovem	X		
Insecta	Orthoptera	Phalangopsidae	*Paraclodes*	jovem	X	1	E
Insecta	Orthoptera	Phalangopsidae	*Phalangopsis*	sp.	X	12	E/P
Insecta	Psocoptera	Epipsocidae	*Epipsocus*	sp.2	X	X	E
Insecta	Psocoptera	Lepidopsocidae		jovem		X	E
Insecta	Psocoptera	Manicapsocidae	*Nothoentomum*	sp.1		X	E
Insecta	Thysanura	Nicoletiidae		sp.1		X	E
Malacostraca	Isopoda	Armadillidae		sp.1	X	X	P
Nematoda				sp.1		X	P
Symphyla		Scutigerellidae		sp.		X	E
Amphibia	Anura			sp.		7	P/A
Mammalia	Chiroptera	Emballonuridae	*Peropteryx*	sp.		X	E
Mammalia	Chiroptera	Furipteridae	*Furipterus*	*horrens*	5	X	E
Mammalia	Chiroptera	Phyllostomidae	*Carollia*	*perspicillata*	5	X	E
